# Vascular smooth muscle cells enhance immune/vascular interplay in a 3-cell model of vascular inflammation

**DOI:** 10.1038/s41598-023-43221-8

**Published:** 2023-09-23

**Authors:** Jolanta Wiejak, Fiona A. Murphy, Pasquale Maffia, Stephen J. Yarwood

**Affiliations:** 1 Institute of Biological Chemistry, Biophysics and Bioengineering, Heriot-Watt University, Edinburgh, EH14 4AS UK; 2 Strathclyde Institute of Pharmacy and Biomedical Sciences, University of Strathclyde, Glasgow, G4 0RE UK; 3 School of Infection & Immunity, College of Medical, Veterinary and Life Sciences, University of Glasgow, Glasgow, G12 8TA UK; 4 School of Cardiovascular & Metabolic Health, College of Medical, Veterinary and Life Sciences, University of Glasgow, Glasgow, G12 8TA UK; 5 Department of Pharmacy, School of Medicine and Surgery, University of Naples Federico II, 80131 Naples, Italy

**Keywords:** Cell biology, Cell signalling

## Abstract

Atherosclerosis is a serious cardiovascular disease that is characterised by the development of atheroma, which are lipid-laden plaques that build up within arterial walls due to chronic inflammatory processes. These lesions are fundamentally attributed to a complex cellular crosstalk between vascular smooth muscle cells (VSMCs), vascular endothelial cells (VECs) and central immune cells, such as macrophages (Mɸs), which promote vascular inflammation. The presence of VSMCs exerts both positive and negative effects during atheroma development, which can be attributed to their phenotypic plasticity. Understanding the interactions between these key cell types during the development of vascular inflammation and atheroma will enhance the scope for new therapeutic interventions. This study aims to determine the importance of VSMCs for shaping the extracellular cytokine/chemokine profile and transcriptional responses of VECs (human coronary artery endothelial cells; HCAECs) to activated lipopolysaccharide (LPS)-stimulated THP1 Mɸs, in a 3-cell model of human vascular inflammation. It is evident that within the presence of VSMCs, enhanced cytokine production was associated with up-regulation of genes associated with vascular inflammation t. Results demonstrate that the presence of VSMCs in co-culture experiments enhanced cytokine production (including CXCL1/GROα, IL-6, IL-8 and CCL2/MCP1) and inflammatory gene expression (including genes involved in JAK/STAT, Jun and NFκB signalling) in HCAECs co-cultured with LPS-stimulated THP1 Mɸs. Our results highlight the importance of VSMCs in immune/endothelial cell interplay and indicate that 3-cell, rather than 2-cell co-culture, may be more appropriate for the study of cellular crosstalk between immune and vascular compartments in response to inflammatory and atherogenic stimuli.

## Introduction

Cardiovascular diseases (CVDs), including atherosclerosis (AS), are the leading cause of death in Western populations^1,2^. The major risk factors of AS development include hyperlipidaemia, hyperglycaemia, insulin resistance, hypertension, aging, obesity, infections and environmental toxins (e.g., cigarette smoke, air pollutants)^1,2^. AS is characterised by the formation of atheroma, a reversable accumulation of lipid-laden plaques made up of fatty deposit lesions that build up on the tunica intima, the inner layer of the arteries^3^. For a long time, AS was considered as a predominantly lipid-driven disease; however, it is now considered as a progressive inflammatory systemic disease of arterial walls and anti-inflammatory strategies are increasingly being considered as therapeutic approaches for the overall prevention and treatment of AS and other CVDs^4–6^.

The pathological mechanisms of AS are complex and include dysfunction of vascular endothelial cells (VECs), macrophage (Mɸ) polarization, vascular inflammation, and immune responses. Endothelial dysfunction is an initial step in the development of AS, is mainly associated with impaired bioavailability of nitric oxide (NO)^7^.

The increased endothelial permeability associated with endothelial dysfunction, allows for the accumulation of circulating LDL cholesterol and its later oxidation, which causes damage to the endothelium and triggers vascular inflammation. Vascular inflammation is detected by increased levels of pro-inflammatory cytokines in the circulation, particularly interleukin 6 (IL-6), which is produced by the activated VECs and VSMCs within the vessel wall^8^. Indeed, IL-6 is strongly associated with the future risk of major cardiovascular events, including atherothrombosis^9–11^, and inhibition of the IL-6 signalling pathway may be beneficial for coronary artery disease, stroke, and arterial fibrillation^12^. IL-6-activated VECs express multiple cytokines, chemokines (e.g. CCL2) and adhesion molecules (VCAM1, ICAM1 and E-selectin), which are involved in monocyte recruitment to the inflamed endothelium, where they differentiate into proinflammatory Mɸs producing reactive oxygen species (ROS)^13,14^. Moreover, IL-6 accelerates inflammatory responses in VSMCs, including upregulation of cyclin D1 and matrix metalloproteinases (MMPs) through activation of JAK/STAT3 signalling pathway and induction of their migration and proliferation^15,16^. Activated VSMCs migrate from the media to the intima, where they form a plaque, together with Mɸs and a range of immune cell subsets, which exacerbates the development of AS and contributes to the subsequent plaque stability^17^.

To understand the complex crosstalk and immune/transcriptional interaction between VSMCs, VECs and Mɸs populations during vascular inflammation, it is necessary to apply a reductionist approach involving in vitro analysis of co-culture cell models^18^. One widely used approach has been to use two-dimensional (2D) cell cultures, involving trans-well culture filters to separate 2-cell co-cultures, which have the advantage of being technically simple and allowing easy isolation of pure cell populations, without the necessity of sorting^18^. An alternative approach to the 2-cell co-culture, recently developed by the Maffia laboratory^19^, is a 3-cell model of vascular inflammation, where activated Mɸs are cultured on the bottom of the plate and VSMCs and VECs are cultured on different sides of a trans-well insert membrane^19^. The system was established using human coronary artery endothelial cells (HCAECs), human VSMCs and differentiated THP-1 Mɸs that had been pre-treated in the presence or absence of lipopolysaccharide (LPS). LPS was used as it is a potent activator of THP-1 Mɸs and encourages an acute inflammatory response through Toll-like receptor 4 (TLR4) signalling, by further inducing the release of pro-inflammatory soluble mediators^20^. Here we apply RNAseq analysis and cytokine profiling to this 3-cell culture model, to investigate the importance of VSMCs in governing the inflammatory crosstalk between activated Mɸs and VECs. Our results highlight the importance of VSMCs in these processes, indicating that 3-cell, rather than 2-cell co-cultures, may be more appropriate to study cellular crosstalk between immune and vascular compartments in response to inflammatory and atherogenic stimuli.

## Results

### Differential gene expression changes in HCAECs in response to co-culture with VSMCs and/or THP-1 Mɸs

**Figure 1:**
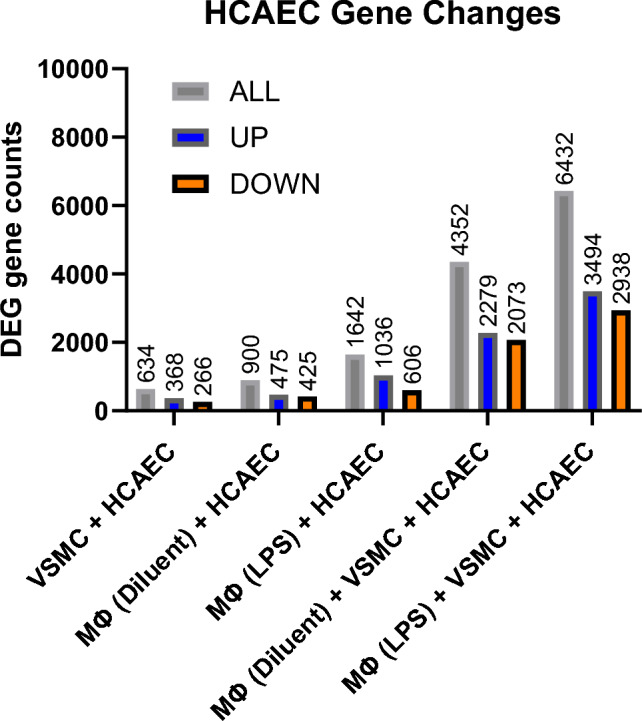
Differential gene expression analysis of endothelial cells co-cultured in the presence or absence of differentiated THP1 Mɸs and/or VSMCs. HCAECs were co-cultured with differentiated THP1 Mɸs (non-stimulated or LPS-stimulated), VSMCs, or a combination of VSMCs and LPS-stimulated or non-stimulated THP1 cells. Following twenty-four hours of co-culture, HCAECs were isolated from culture. Total RNA was then extracted from HCAECs and processed for RNAseq, as described in “Materials and methods”. The number of differentially expressed genes (DEG gene counts; including up-regulation and down-regulation) for each treatment comparison combination is shown here in a histogram. The comparison group software DESeq2 was used for analysis using a significance threshold (p-value) of ≤ 0.05 for differential gene screening.

**Figure 2:**
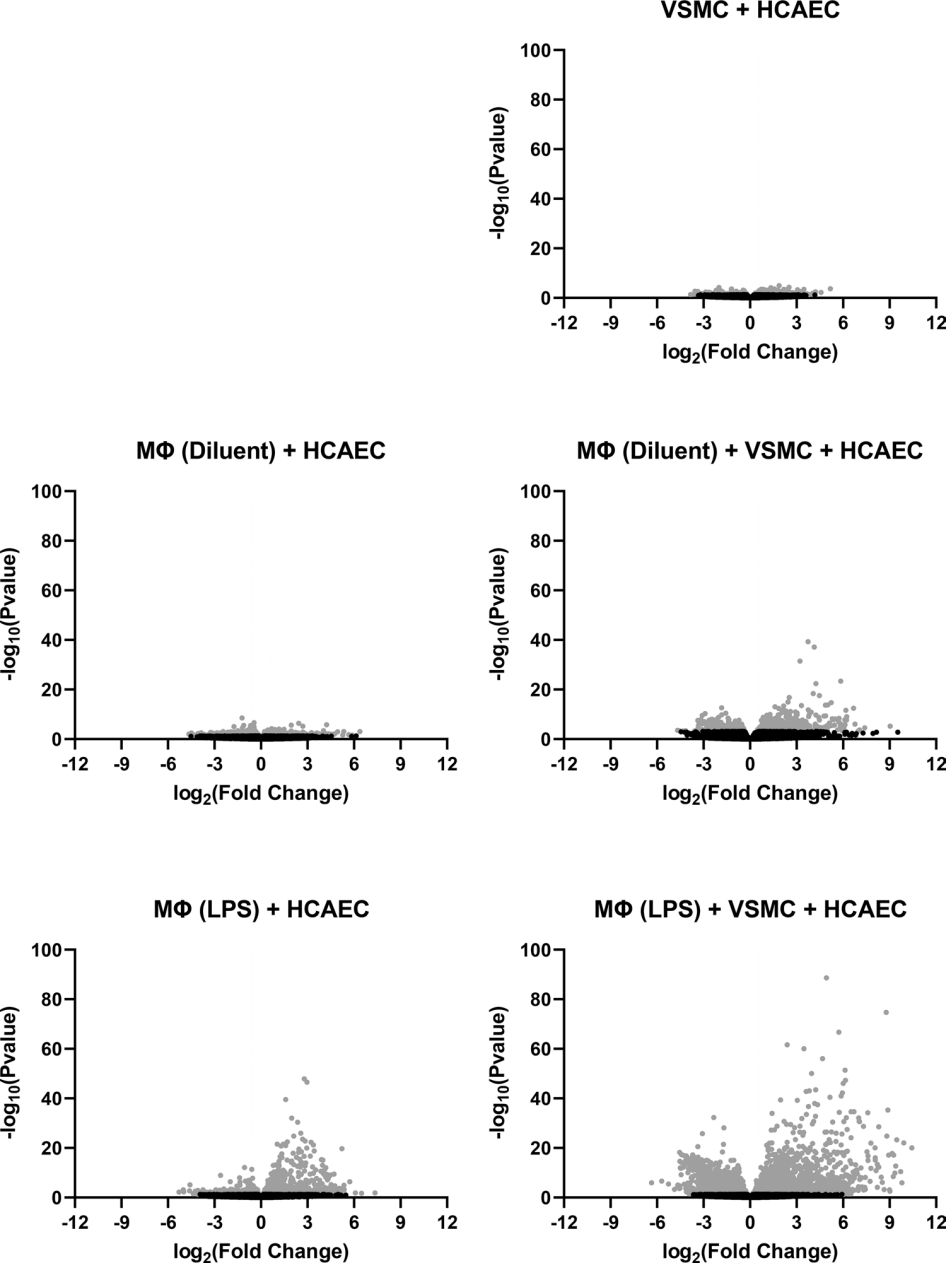
Distribution of differentially expressed genes in HCAECs cultured in the presence or absence of differentiated THP1 Mɸs and/or VSMCs. The differentially expressed genes identified by RNASeq in the comparison groups described in Fig. 1 were plotted as volcano plots to infer the overall distribution of differentially expressed genes in each treatment comparison group. In the figures, the x-axis shows the fold change in gene expression between different samples, and the y-axis shows the statistical significance of the differences. Significant gene expression changes (p ≤ 0.05; either up- or down-regulated) are shown in *light grey*. Non-significant protein changes are shown in black. The horizontal, dashed lines indicate the threshold line for differential gene screening criteria (p ≤ 0.05 and > twofold change).

The complex cellular interactions between VSMC, VECs and immune cells are central to the development of degenerative lesions associated with atherogenesis. To investigate the importance of these interaction we carried out RNAseq analysis of HCAECS (Human Coronary Artery Endothelial Cells) with different combinations of human VSMCs and/or PMA-differentiated THP-1 Mɸs that were then treated in the presence or absence of LPS, to promote production of cytokine and chemokines. The protocols for cell culture, RNA extraction, RNAseq and bioinformatics are described in the Methods section. The resulting differentially expressed gene (DEG) counts in HCAECs, in response to the different culture conditions applied, are shown in Fig. 1 and the distribution of statistically significant gene expression changes are shown in Fig. 2 and recorded in the Supplementary Data sheet. As expected, it was observed that the presence of LPS-pre-treated THP1 Mɸs, or non-stimulated THP-1 Mɸs, influenced gene expression patterns in co-cultured HCAECs, by promoting both gene up-regulation and down-regulation (Figs. 1 and 2). Accordingly, the presence of LPS-pre-treated THP1 Mɸs promoted 1642 gene expression changes in HCAECs (1036 up- and 606 down-regulated), compared to 900 gene expression changes (475 up- and 425 down-regulated) induced by non-stimulated THP1 Mɸs (Fig. 1). Notably, it was found that co-culture with VSMCs, exerted a synergistic effect on gene expression in HCAECs (Figs. 1 and 2).The presence of VSMCs alone was found to regulate the expression of 634 genes in HCAECs (368 up- and 266 down-regulated), whereas in the presence of both VSMCs plus non-stimulated THP1 Mɸs, this was increased to 4352 genes, compared to 900 in the absence of VSMCs (Fig. 1). Similarly, a combination of VSMCs plus LPS-pre-treated THP1 Mɸs provoked 6432 gene changes in HCAECs (3494 up- and 2938 down-regulated), compared to 1642 gene changes, in the absence of VSMCs (Fig. 1). Overall, maximal gene expression changes in HCAECs are achieved through co-culture with VSMCs in the presence of LPS-pre-treated THP1 Mɸs.

### Categorisation of gene expression changes in HCAECs

**Figure 3:**
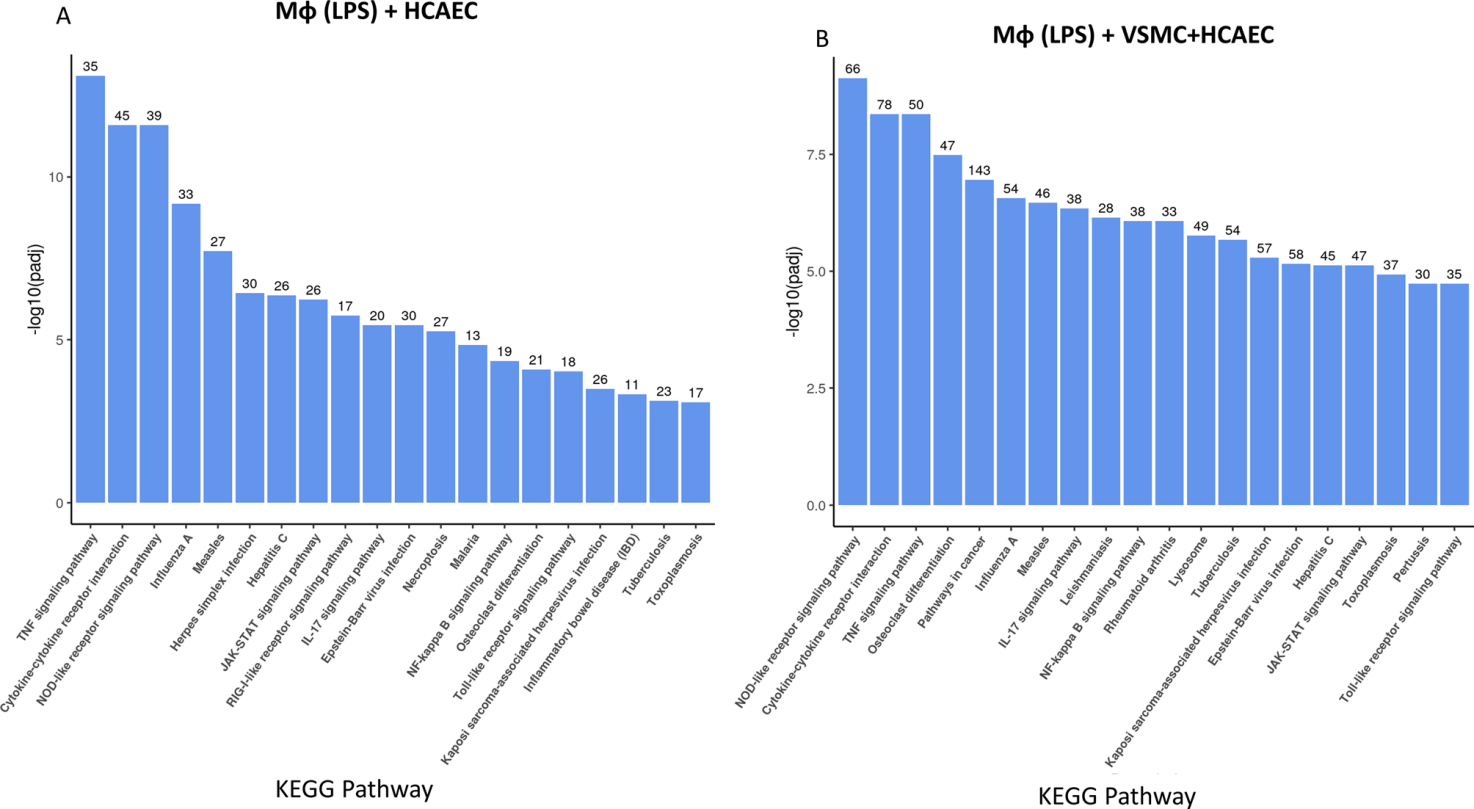
KEGG analysis of gene expression changes in HCAECs incubated with LPS-stimulated differentiated THP1 Mɸs, in the presence or absence of VSMCs. Upregulated gene expression from HCAECs, co-cultured with LPS-pre-treated differentiated THP1 Mɸs, in the absence (**A**) or presence (**B**) of VSMCs, were further analysed to identify common biological functions associated with gene expression changes by comparison with the Kyoto Encyclopaedia of Genes and Genomes (KEGG) database^54,55^. From the KEGG enrichment results, the most significant 20 KEGG pathways were selected for display as a histogram, with the x-axis indicating the KEGG pathway, and the y-axis represents the significance level of pathway enrichment. The number of genes associated with each KEG pathway is indicated at the top of each bar.

We carried out KEGG pathway analysis of the RNAseq data to classify the gene expression changes detected in HCAECs into known functional groups (Fig. 3). We compared the gene expression changes in HCAECs influenced by LPS-pre-treated THP1 Mɸs in the presence (Fig. 3B) or absence (Fig. 3A) of VSMCs. We found that, in both treatment groups, the top 3 KEGG pathways contained genes involved in signalling pathways associated with inflammatory responses in HCAECs, notably the TNF (Tumour Necrosis Factor) signalling, cytokine receptor interaction and NOD-like receptor signalling pathways were highly represented (Fig. 3). Importantly, the number of genes associated with each of these signalling networks was greater in the presence of VSMCs (Fig. 3B) compared to the absence of VSMCs (Fig. 3A).

**Figure 4:**
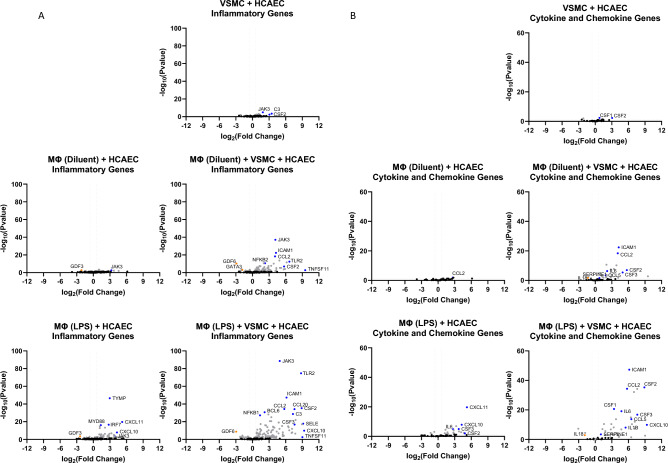

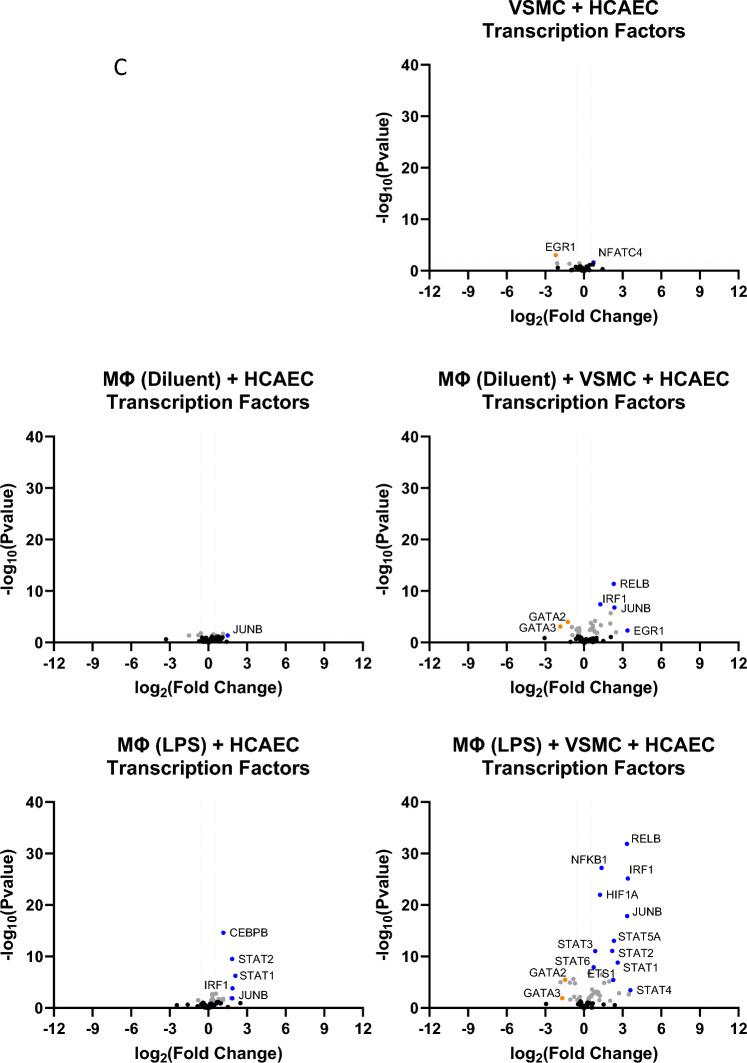
Inflammatory gene expression in HCAECs in the presence or absence of THP1 Mɸs and/or VSMCs. RNA was extracted from HCAECs that had been cultured in the presence or absence of differentiated THP1 Mɸs (LPS stimulated or non-stimulated) and/or VSMCs as described in “Materials and methods”. RNAseq was then carried out and the resulting gene expression changes of selected genes associated with (**A**) inflammation, (**B**) cytokines and chemokines or (**C**) transcription factors (gene lists are found in Supplementary Data 1) are shown here as volcano plots. Significant gene changes (p < 0.05) are shown in *light grey* whereas non-significant gene changes are shown in *black*. Significant changes in selected inflammatory gene expression are indicated in *red* (for up-regulation) or *green* (for down-regulation).

To further characterise the gene expression changes in HCAECs that were amplified by co-culture with VSMCs, we used volcano plots of the RNAseq data to identify genes associated with inflammation (Fig. 4A), cytokines and chemokines (Fig. 4B) and transcription factors (Fig. 4C). We noted that genes known to be involved in inflammatory responses, including JAK/STAT, Jun and NFκB signalling routes (Fig. 4A and C), and soluble mediators, including ICAM1, CCL2/MCP1, IL-6 and IL1β (Fig. 4B), were induced in the presence of LPS-pre-treated THP1 Mɸs and further amplified in the presence of VSMCs. This suggests, that VSMCs can amplify the inflammatory gene response in HCAECs, through amplification of immune/vascular interplay.

### Cytokine/chemokine array analysis of secretory factors in co-culture

**Figure 5:**
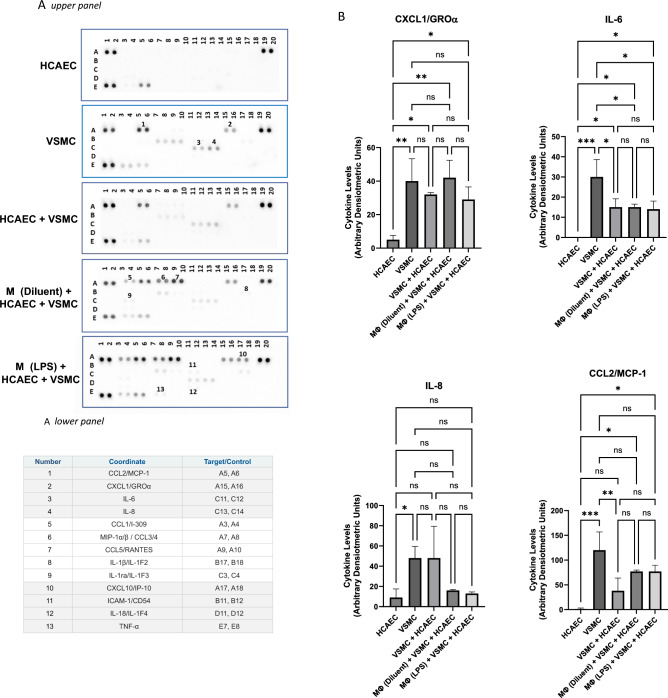
Cytokine array profiling of soluble factors from cultures of HCAECs in the presence or absence of THP1 Mɸs and/or VSMCs. HCAECs and VSMCs were cultured in isolation or co-cultured with differentiated THP1 Mɸs as shown and described in “Materials and methods”. The growth medium from individual cultures was then overlayed on filter membranes spot-printed with antibodies, in the *upper panel* of (**A**), and then developed as described in the “Materials and methods”. The table in the *lower panel* in (**A**) describes the spot intensities that were induced during the different treatments applied (non-constitutive) and the corresponding numbers refer to the spot array in the *upper panel*. The full list of cytokine antibodies spotted on each array is shown in Supplementary Data 2. Changes in spot intensity specifically identified in cultures of VSMCs alone are shown in (**B**), with significant changes in cytokine levels being indicated; *p < 0.05, **p < 0.01 and ***p < 0.001 (n = 3). Non-significant changes are also shown (ns).

Our hypothesis is that VSMCs enhance immune/vascular interplay, in this case between THP1 Mɸs and HCAECs in co-culture, leading to enhancement of inflammatory gene expression in HCAECs. In terms of the mechanisms by which VSMCs could amplify gene responses in HCAECs, it is likely that VSMCs produce additional soluble factors, in addition to those produced by Mɸs, that amplify inflammatory signalling in HCAECs. To identify these potential factors, we used an array printed with antibodies to a range of known cytokines and chemokines (Fig. 5A), to identify soluble factors in supernatants from co-culture experiments. As expected, maximal secretion of cytokines and chemokines was observed in supernatants from co-cultures of HCAECs, VSMCs, and LPS-pre-treated THP1 Mɸs, including key inflammatory mediators ICAM1, TNFα and IL1β, amongst a range of chemokines, including CCLs 1–5 (Fig. 5A and Supplementary Data 2). However, we also found significant secretion of CXCL1/GROα, IL-6, IL8 and CCL2/MCP1 in co-cultures of VSMCs and HCAECs and in cultures of VSMCs alone, in the absence of THP1 Mɸs (Fig. 5B), indicating that these factors may play a role in enhancing inflammatory responses in HCAECs, independently of those produced by co-culture with THP1 Mɸs.

## Discussion

The aim of this study was to compare the complex interaction between immune cells and the transcriptional responses of vascular endothelial cells (VECs) in the presence or absence of VSMCs, using an experimental model of vascular inflammation^19^. Despite our understanding that crosstalk between VECs and immune cells underlies the development of AS, most cell-based studies use mono-culture techniques. The advantage of the 3-cell culture protocol used here, which consists of HCAECs, VSMCs and LPS- -treated THP1 Mɸs, has the advantage of being technically simple and allows the isolation of pure cell population from co-culture without the need for cell sorting^19^. This 3-cell system has been used to demonstrate the interaction between VECs, VSMCs and THP1 Mɸs and identified soluble mediator profiles that have yet to be observed in 2-cell cultures^19^. Most notably, in the current study we found that VSMCs appeared to play an essential role in this communication by enhancing gene regulation in HCAECs, brought about by co-culture with PMA-differentiated, LPS-pre-treated THP1 Mɸs (Figs. 1 and 2). More recent 3-cell models of atherosclerosis rely on the use of non-differentiated THP1 monocytes^21^, so it is questionable whether the levels of inflammation induced in these systems are fully representative of immune/vascular interplay in native atheroma.

We found that the presence of VSMCs in co-culture with THP1 Mɸs, led to a dramatic induction of many genes in HCAECs involved in vascular inflammation (Fig. 3), including a striking induction of JAK3 (Fig. 4A) expression, which is a downstream kinase that is activated bymany pro-inflammatory cytokines, and is a known drug target for the treatment of inflammation-driven pathologies like rheumatoid arthritis, psoriasis and inflammatory bowel disease^22^. JAK3 is an essential enzyme for the immune system and is primarily involved in receptor-mediated signal transduction, through the signal transducer and activator of the transcription (STAT) protein family^23^, of which STATS 1–6 were significantly upregulated in the presence of LPS- -treated THP1 Mɸs and VSMCs (Fig. 4C). STATs play key roles in lipid metabolism and proinflammatory response signals in the expression regulation of adhesion molecules, chemokines and subsequent atherogenesis^24–27^. This further supports the complex interplay between immune transcription factor genes and VSMCs within atherosclerosis.

NFKB1, RELB and IRF1 were also identified as inflammatory transcription factors that show enhanced upregulation in HCAECs in the presence of VSMCs (Fig. 4C). NFKB1 is the most highly expressed transcription factor in macrophages and is essential to the mechanisms of atherosclerosis development^28^. Furthermore, the expression of IRF1, has been found to play a predominant role in the formation and stability of lesions and contributing to foam cell formation, which is also advanced by VSMCs^29^, resulting in necrosis, plaque rupture and subsequent thrombosis^30^. Hence, although VSMCs have been viewed as plaque-stabilizing, several studies demonstrate that VSMCs display a range of different phenotypes in vivo, including functions normally associated with macrophages, foam cells, osteochondrogenic cells, myofibroblasts and mesenchymal stem cells, that are potentially pathogenic^29^. Indeed, using cytokine arrays to detect soluble factors in the cell culture model presented here (Fig. 5), we find that co-culture with VSMCs enhances the production of chemotactic factors (CXCL1/GROα, CCL2/MCP1 and IL-8), which are key regulators of mononuclear cell recruitment to atherosclerotic plaques^31,32^, and IL-6, which is normally associated with chronic inflammation and autoimmunity, including atherosclerosis^10,33^.

To fully understand the role VSMCs in immune-vascular interplay it is important to understand the soluble factors produced by VSMCs during vascular inflammation and how these are regulated. As well as secretion of interleukins, chemokines and cytokines (including TGF-β, IL-1, IL-6, and TNF-α^34^) VSMCs play a significant role in regulating vascular tone, blood pressure, and other physiological processes, including inflammation^35^, through the production of classical soluble regulators, including vascodilators (eg prostaglandins PGI2 and PGE1^36^) and vasoconstrictors (eg thromboxanes^37,38^, endothelin^39^ and angiotensin II^34^), as well as growth factors (eg PDGF and thrombin^34^), matrix metalloproteinases^40^ and adhesion molecules (eg ICAM1^35^). Dysregulation of their production or signalling can contribute to various cardiovascular diseases, including hypertension, vascular remodelling, and atherosclerosis^35^.

In addition to these classical factors, non-coding RNAs (ncRNAs) appear to play a diverse and significant role in the regulation of various cellular processes, including those within VSMCs. These molecules do not code for proteins but instead have regulatory functions at the RNA level. For example, in VSMCs, microRNAs (miRNAs) are involved in controlling processes such as VSMC proliferation, migration, and differentiation, as well as extracellular matrix remodelling^41^. Dysregulation of miRNA expression in VSMCs has been linked to vascular diseases, including atherosclerosis and restenosis^42,43^. Long Non-coding RNAs (lncRNAs) are the largest population of ncRNAs and act as competing endogenous RNAs (ceRNAs) by sequestering miRNAs and influencing the expression of miRNA target genes^41^. Some lncRNAs in VSMCs have been found to modulate processes such as VSMC phenotype switching (from contractile to synthetic), vascular inflammation, and cell proliferation^44–47^. Circular RNAs (circRNAs) are a type of ncRNA that forms a closed loop structure and can act to sequester miRNAs, thus regulating the availability of miRNAs for target mRNAs. CircRNAs in VSMCs may impact processes like VSMC proliferation, migration, and extracellular matrix regulation^48^. The mechanism of actions of ncRNAs is diverse but, particularly relevant to the results here is their ability to regulate pro-inflammatory signalling, eg through the STAT3 (Signal Transducer and Activator of Transcription 3) and NFkB pathways, to regulate the production of pro-inflammatory cytokine production, including IL-6, CCL2 and TNFα^41,49^. Overall, ncRNAs produced by VSMCs play a significant role in regulating key processes related to vascular physiology and pathology. Their dysregulation can contribute to vascular diseases, making them important targets for research and potential therapeutic interventions. Therefore, while VSMCs are central to the components of arterial wall structural and regulate vascular homeostasis as well as other processes through peripheral resistance, they are present in all stages of atheroma development^29^. This study suggests that due to their presence they can influence the transcriptional responses of VECs which further promotes atherosclerosis associated genes and mechanisms associated with disease development. Therefore, potentially targeting the phenotypic changes of VSMCs could prevent the advancement of plaque development or increase plaque stability. Moreover, to fully understand the impact of immune-vascular interplay it is important that VSMCs be included in experimental systems, as demonstrated with the 3-cell model of atheroma presented here and previously^19^.

## Materials and methods

### Materials

Cryopreserved human coronary artery endothelial cells (HCAEC; 500,000 cells; Cat# C-12221), human coronary artery smooth muscle cells (VSMC; 500,000 cells; Cat# C-12511), ready to use endothelial cell growth medium MV2 (Cat# C-39226 for supplement mix and Cat# C22022B for Media) and ready to use smooth muscle cell medium 2 (Cat# C-39267 for supplement mix and Cat# C22062B for media) were purchased from PromoCell GmbH (Heidelberg, Germany). THP-1 cells were obtained from the American Type Culture Collection (ATCC; Cat# TIB-202). Heat-inactivated foetal bovine serum (FBS; Cat# 758093) was purchased from Greiner Bio-One (Gloucestershire, UK). Phorbol 12-myristate 13-acetate; (PMA; Cat# P8139-1MG or P8139-5MG), lipopolysaccharide from Escherichia coli (LPS; Cat# L4391) and Corning™ Transwell™ Multiple Well Plate with Permeable Polyester Membrane Inserts (0.4 µm pore; 6 well format; Cat# CLS3450-24EA) were from Merck Life Sciences UK Limited (Glasgow, Scotland). Roswell Park Memorial Institute (RPMI) 1640 Medium containing L-Glutamine (Cat# 21875-034) was purchased from ThermoFisher Scientific (Renfrew, Scotland).

### Cell culture

Cultures of human coronary artery endothelial cells (HCAECs) and human coronary artery smooth muscle cells (VSMCs) were maintained and passaged in endothelial cell growth medium MV2 and smooth muscle cell medium 2, respectively, as previously described^19^. THP-1 monocytes/macrophages were maintained in RPMI media supplemented with 10% (v/v) foetal bovine serum, l-glutamine, and Penicillin/Streptomycin (Merck Life Sciences UK Limited (Glasgow, Scotland) and differentiated into Mɸs when needed, by incubation with the phorbol ester, phorbol-12-myristate-13-acetate (PMA), as previously described^19^.

### Co-culture of cells

VSMCs were seeded onto the basal side of an upturned transwell insert (1.8 × 10^5^ cells in 500 µl) and allowed to adhere for 6 h at 37 °C. Transwell inserts were then inserted into individual wells of 6-well plates and incubated in 1.5 ml media for 72 h at 37 °C until confluent. This step was omitted for experiments done in the absence of VSMCs. The following day, THP-1 cells were seeded onto 6 well plates (1 × 10^6^ cells per well) and then differentiated with the addition of PMA (100 ng/ml) for 24 h at 37 °C, as previously described^19^. On the same day, HCAECs (1.8 × 10^5^ cells per well) were seeded onto the apical side of transwell inserts (with or without VSMCs on the basal surface), and incubated at 37 °C. Next day, the media of THP-1 cells was replaced with fresh RPMI, containing 10% (v/v) FBS and then rested for a further 24 h at 37 °C. The following day, the THP-1 cells were exposed to lipopolysaccharide (LPS) (100 ng/ml) in 2 ml media per well for 2 h, incubated at 37 °C. For certain experiments, the addition of LPS was omitted at this stage and cells were stimulated with diluent alone. After 2 h, the media was removed and replaced with fresh media and transwell inserts (carrying monolayers of HCAECs plus or minus VSMCs) were transferred to wells containing LPS-stimulated or diluent-stimulated THP-1 cells. Fresh media (1 ml) was then added to the apical side of the transwell and cell co-cultures were incubated at 37 °C for a further 20 h. Following this, 1 ml of cell supernatant from the basal side of each transwell was removed and stored at -20 °C for cytokine profiling. In addition, cells were washed with ice-cold sterile phosphate buffered saline (PBS) and then 500 µl of ice-cold, sterile PBS was added to the apical side of each transwell and HCAECs were harvested by scrapping with rubber-tipped plunger from sterile 1 ml syringe. Cell suspensions of HCAECs from duplicate treatment wells were pooled. HCAECs were then centrifuged and resuspended in 350 µl RTL lysis buffer from a RNeasy Mini Kit (Qiagen, Manchester, UK) and stored at − 20 °C.

### RNA sequencing

Total RNA was isolated from co-cultured HCAECs using a RNeasy Mini Kit (Qiagen, Manchester, UK), according to the manufacturer's protocol. RNA concentration was determined using NanoDrop 1000 Spectrophotometer (Thermo Fisher Scientific, Paisley, UK). RNA sample quality control, RNA library preparation, library quality control, Illumina library sequencing, data quality control and bioinformatics analysis were then carried out by Novogene UK (Cambridge, UK). In brief, raw reads of FASTQ format were firstly processed through in-house Perl scripts. In this step, clean data (clean reads) were obtained by removing reads containing adapter, reads containing poly-N, and low-quality reads from raw data^50^. All the downstream analyses were based on the clean data with high quality. Paired-end clean reads were aligned to the human genome using Hisat2 v2.0.5^51^. Then, the abundance of each transcript was quantified using FeatureCounts v1.5.0-p3^52^. Differentially expressed gene (DEGs) analysis was performed using the DESeq2 package^53^ and gene expression, compared to HCAECs alone, was normalized using relative-log-expression (RLE). Genes with an adjusted p-value < 0.05 were assigned as being differentially expressed. Metascape (http://metascape.org) was employed to perform gene enrichment and functional annotation analyses by sourcing the Kyoto Encyclopaedia of Genes and Genomes (KEGG) Pathway^54,55^. The profileR package^56^ was used to test the statistical enrichment of differential expression genes in KEGG pathways.

### Cytokine array

Media from single- double and triple-cell cultures were centrifuged at 10,000*g* for 10 min to remove particulates. The remaining supernatant was then assessed for the detection of 36 different cytokines and chemokines using Proteome Profiler Human Cytokine Array Kit (R&D Systems) according to the manufacturer's instructions. Briefly, 1 mL of medium was mixed with 0.5 mL of Array Buffer 4 and 15 µL of Detection Antibody Cocktail and incubated at room temperature for 1 h. At the same time array membranes were blocked in 2 mL of Array Buffer 4 (1 h, RT) and then reaction mixture was added to the appropriate membranes for overnight exposure (4 °C on rocking platform). After the incubation and washing (3 × 10 min) in 1× Wash Buffer, each membrane was exposed to Streptavidin-HRP solution (diluted 1:2000 in Array Buffer 5) for 30 min at RT and then activated by Chemi Reagent Mix. The chemiluminescence signal in the form of dark dots on the membranes was detected using Fusion FX7 camera platform (Vilber, Collégien, France). The optical density of dots was measured by ImageJ software (National Institutes of Health, Bethesda, USA). Cytokine levels were expressed as a % of the positive reference spots for each array.

### Supplementary Information

Supplementary Information 1.

Supplementary Information 2.

## Data Availability

All data generated or analysed during this study are included in this published article and in supplementary data file 1.
