# Supplementary material for: Vascular smooth muscle cells enhance immune/vascular interplay in a 3-cell model of vascular inflammation
Source: Sci Rep. 2023 Sep 23;13:15889. doi: 10.1038/s41598-023-43221-8 (PMC10517978; doi:10.1038/s41598-023-43221-8)
Supplement: Supplementary file 2 — Supplementary Information 2. [file 41598_2023_43221_MOESM2_ESM.pptx]

## Slide 1
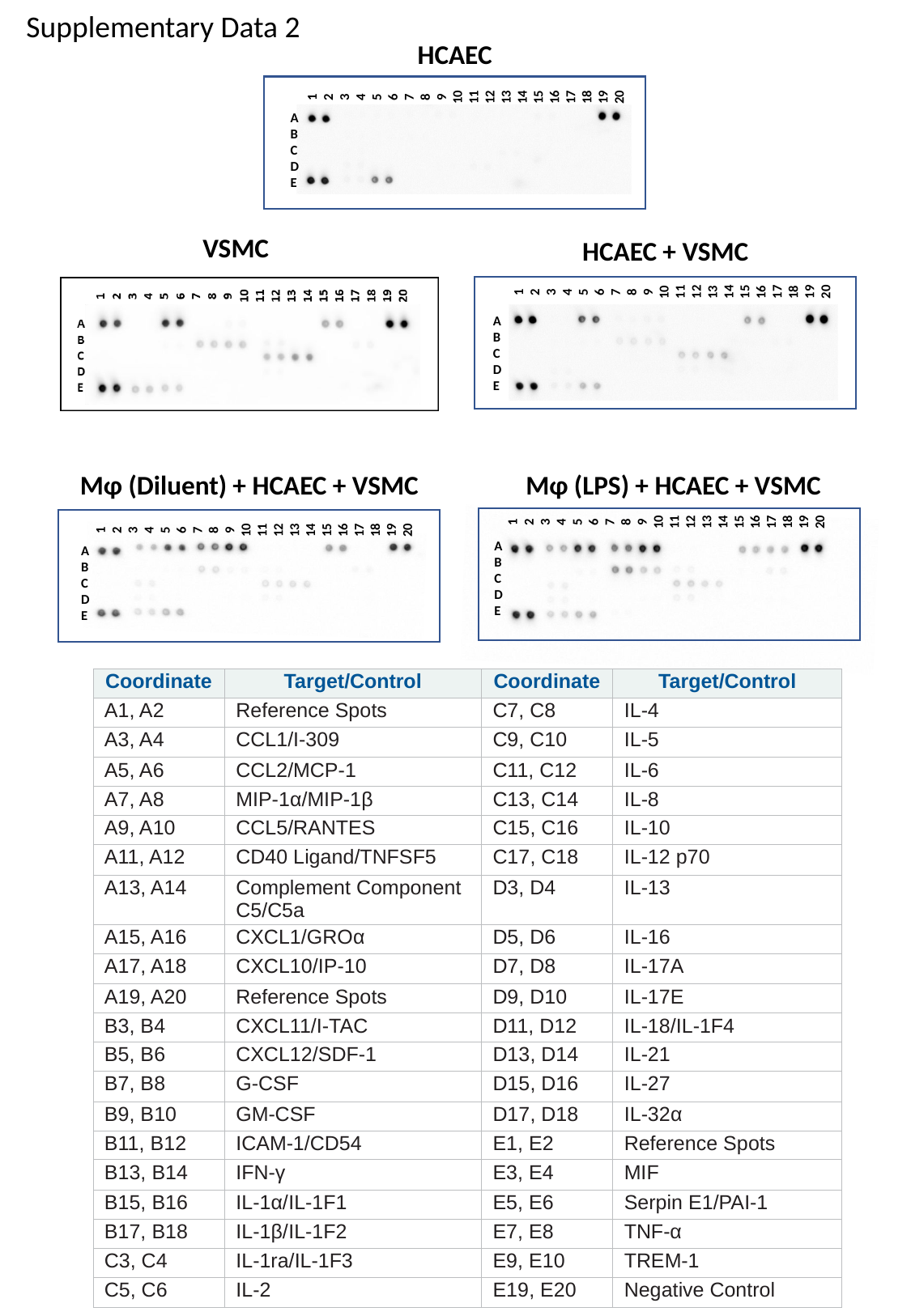

Supplementary Data 2
HCAEC
1
2
3
4
5
6
7
8
9
10
11
12
13
14
15
16
17
18
19
20
A
B
C
D
E
VSMC
HCAEC + VSMC
1
2
3
4
5
6
7
8
9
10
11
12
13
14
15
16
17
18
19
20
A
B
C
D
E
Mɸ (Diluent) + HCAEC + VSMC
1
2
3
4
5
6
7
8
9
10
11
12
13
14
15
16
17
18
19
20
A
B
C
D
E
Mɸ (LPS) + HCAEC + VSMC
1
2
3
4
5
6
7
8
9
10
11
12
13
14
15
16
17
18
19
20
A
B
C
D
E
| Coordinate | Target/Control | Coordinate | Target/Control |
| --- | --- | --- | --- |
| A1, A2 | Reference Spots | C7, C8 | IL-4 |
| A3, A4 | CCL1/I-309 | C9, C10 | IL-5 |
| A5, A6 | CCL2/MCP-1 | C11, C12 | IL-6 |
| A7, A8 | MIP-1α/MIP-1β | C13, C14 | IL-8 |
| A9, A10 | CCL5/RANTES | C15, C16 | IL-10 |
| A11, A12 | CD40 Ligand/TNFSF5 | C17, C18 | IL-12 p70 |
| A13, A14 | Complement Component C5/C5a | D3, D4 | IL-13 |
| A15, A16 | CXCL1/GROα | D5, D6 | IL-16 |
| A17, A18 | CXCL10/IP-10 | D7, D8 | IL-17A |
| A19, A20 | Reference Spots | D9, D10 | IL-17E |
| B3, B4 | CXCL11/I-TAC | D11, D12 | IL-18/IL-1F4 |
| B5, B6 | CXCL12/SDF-1 | D13, D14 | IL-21 |
| B7, B8 | G-CSF | D15, D16 | IL-27 |
| B9, B10 | GM-CSF | D17, D18 | IL-32α |
| B11, B12 | ICAM-1/CD54 | E1, E2 | Reference Spots |
| B13, B14 | IFN-γ | E3, E4 | MIF |
| B15, B16 | IL-1α/IL-1F1 | E5, E6 | Serpin E1/PAI-1 |
| B17, B18 | IL-1β/IL-1F2 | E7, E8 | TNF-α |
| C3, C4 | IL-1ra/IL-1F3 | E9, E10 | TREM-1 |
| C5, C6 | IL-2 | E19, E20 | Negative Control |
